# Far field superlensing inside biological media through a nanorod lens using spatiotemporal information

**DOI:** 10.1038/s41598-021-81091-0

**Published:** 2021-01-21

**Authors:** Mohamad J. Hajiahmadi, Reza Faraji-Dana, Anja K. Skrivervik

**Affiliations:** 1 Center of Excellence on Applied Electromagnetic Systems, School of Electrical and Computer Engineering, College of Engineering, University of Tehran, Tehran, Iran; 2 Microwave and Antenna Group (MAG), École Polytechnique Fédérale de Lausanne (EPFL), Lausanne, Switzerland

**Keywords:** Optics and photonics, Optical physics

## Abstract

Far field superlensing of light has generated great attention in optical focusing and imaging applications. The capability of metamaterials to convert evanescent waves to propagative waves has led to numerous proposals in this regard. The common drawback of these approaches is their poor performance inside strongly scattering media like biological samples. Here, we use a metamaterial structure made out of aluminum nanorods in conjunction with time-reversal technique to exploit all temporal and spatial degrees of freedom for superlensing. Using broadband optics, we numerically show that this structure can perform focusing inside biological tissues with a resolution of λ/10. Moreover, for the imaging scheme we propose the entropy criterion for the image reconstruction step to reduce the number of required optical transducers. We propose an imaging scenario to reconstruct the spreading pattern of a diffusive material inside a tissue. In this way super-resolution images are obtained.

## Introduction

Manipulation of light from far field in nanometer scale resolution is a topic of broad interest to physicists and engineers alike. Super-resolution imaging and its reversed process for writing may find applications in biological tissue imaging^1^, photonic crystal fabrication^2^, laser therapy^3^ and art preservation^4^. However, in the presence of noise the resolution of imaging is finite and we cannot go beyond a certain limit. In conventional optical devices the diffraction limit is at about half a wavelength. Using wave optics, it can be shown that one of the approaches for imaging below this resolution is to measure the evanescent waves, which vanish exponentially away from the object^5^. The information carried by these decaying waves can help in superlensing.

The need for super-resolution imaging and focusing system especially inside biological samples has led to various efforts on breaking the diffraction limit in the past decades. First, near field scanning optical microscopy (NSOM)^6^ was introduced in which evanescent waves are measured by point-by-point scanning system using a near field probe. Although NSOM is a high-resolution technique, it is difficult to operate in a noninvasive mode and has a low imaging depth. Fluorescence-based far field optical microscopy approaches including stimulated emission depletion (STED)^7^, photo-activated localization microscopy (PALM or FPALM)^8^ and stochastic optical reconstruction microscopy (STORM)^9^ opened new opportunities for biological applications and realized resolution of tens of nanometers, substantially lower than the wavelength of light. These approaches are based on the ability to control subsets of fluorophores that are forced to be non-fluorescent and other subsets that are forced to be capable of fluorescence^10^. Although these super-resolution techniques have successfully demonstrated their ability to resolve nanometer scale objects, each has its own limitations. STED and similar methods are based on nonlinear optical effects, and typically require the use of high-intensity pulsed lasers, which can lead to invasive lensing. PALM and STORM are highly sensitive to contrast of fluorescent probes and background noise^11^. Also, most published works in fluorescence-based super-resolution imaging methods only demonstrated imaging of fixed biological samples and continuing effort is needed to make these methods applicable to study biological processes and structural changes in living samples^12^.

On the other hand, many proposals of hyperlenses^13–16^ and structured illumination methods^17–19^ were presented. Far field imaging systems based on these structures are made of metamaterials supporting evanescent modes which can resolve fine structures at nanometer accuracy^20,21^. However, manufacturing these lenses leads to a huge complexity. Another reported structure for super-resolution imaging is based on SiO_2_ microspheres to realize a superlens^22^. In this method, the reconstructed images by each microsphere are used to form the complete image. The main drawback is the microscopy of large samples. Super-oscillatory lenses^23,24^ can also be used for sub-diffraction imaging. Although this is a high-resolution method, the object must be scanned and this can be time consuming especially for imaging large samples. Recently, gradient metasurfaces have been proposed to extract information from large propagating wave vectors in order to go beyond the diffraction limit^25,26^. Such monochromatic approaches seem limited, since all spatial information propagating away from an object is mixed in a unique wave field. From spatiotemporal degrees of freedom point of view, this results in the loss of a considerable part of the information. In addition, in the optical band, the loss characteristics of the materials are somewhat weak and storing all the information in *only* one unique field is not sensible.

It was recently demonstrated that, due to Fano interferences^27^, a cluster of resonators arranged on a sub-wavelength scale supports wave fields that oscillate on a scale much finer than the free space wavelength and forms a lens in the near field of an object^28,29^. These structures are used to convert evanescent waves scattered by sub-wavelength features into propagating waves resulting in lensing from far field with a resolution beyond the diffraction limit. In addition to far field superlensing, when an object is illuminated by a broad range of energies, the resonant structure creates evanescent waves that decompose in modes of the system with a unique set of phases and amplitudes, so that all spatial information is allowed to propagate. In Refs.^27^ and^30^, respectively, a resolution of $\lambda /25$ and $\lambda /23$ are demonstrated for focusing inside free space.

Refractive index inhomogeneities of the biological tissues cause light to be strongly scattered and thus make a huge obstacle to the imaging and focusing of light^31^. For a long time, scattered light has been viewed as a source of noise. However, time-reversal technique^32^, which refers to time-reversal invariance of Maxwell’s Equations, have changed this view. It has been shown that wave disorder and multipath propagation in the medium can be turned into an ally for finer resolution imaging and better focusing in a highly scattering medium than in a homogeneous medium^33^. First tested in the microwave band^34^, that concept has also been applied to audible acoustics inside disordered bubbly media^35^ and in the light region^30,36^.

In this paper, we propose a structure of optical metamaterial made out of plasmonic nanoparticles with four optical transducers for the far field manipulation of light in nanometer scale. We present a numerical investigation of using polychromatic light in conjunction with time-reversal technique, which shows focusing and imaging inside biological samples with a resolution far beyond the diffraction limit. In the following sections, we first explain the proposed structure and capability of converting evanescent waves to propagating waves. After that, super-resolution focusing is presented and it is shown that time-reversal focusing inside a scattering medium results in a higher resolution and a clearer field distribution inside the medium compared to the homogeneous medium. In the next section, imaging inside a biological sample is investigated for rigid and diffusive material. Also, the minimum entropy criterion is introduced for imaging with low number of transducers. Finally, different aspects of the proposed approach are discussed.

## Metamaterial structure

### Unit cell design

**Figure 1:**
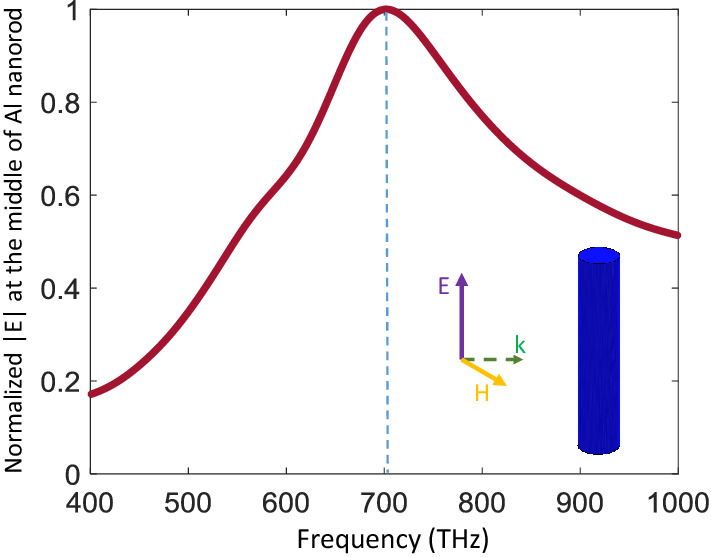
Electric field intensity at the center of a 100 nm long Al rod with 20 nm diameter, illuminated by a linearly polarized plane wave.

In this study, we focus on a plasmonic lens made of a square lattice of sub-wavelength nanoparticles, thus the first step is to design the resonant unit cell. Here, we use aluminum nanorods to realize the resonance at visible wavelength. According to recent developments in fabrication technologies of aluminum structures^16,37^, we set the height and diameter of the rod to 100 and 20nm, respectively. Figure 1 depicts the field recorded at the center of a single lossy aluminum rod using the material property given in Ref.^38^ excited by a linearly polarized plane wave. The resonance peak appears at about 700 THz.

### Resonant nanorod structure

**Figure 2:**
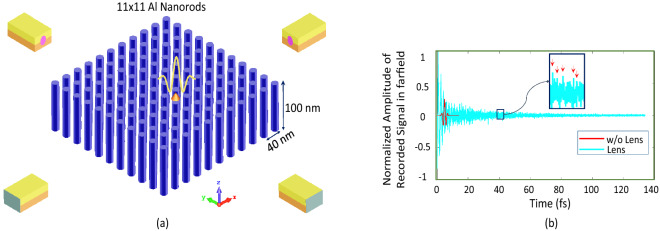
Nanorod lens (**a**) 11 × 11 square lattice of Al nanorod with four optical transducers. (**b**) recorded field in the far field of structure, when a short 10-fs pulse illuminates the lens in comparison to the absence of lens.

To build the structure, we put a collection of parallel nanorods in a square periodic lattice. We numerically study a medium consisting of $N\times N$ (N=11) aluminum nanoparticles with a period of *s* (40 nm) center to center of the rods in transverse directions (Fig. 2a). Now, according to the theory of wire media^39,40^, the structure can be described as a cluster of oscillators with a set of eigenmodes and eigenfrequencies^27^. In other words, any illuminated object placed on top of this nanorod structure, makes evanescent waves that decompose onto the modes of the system with a unique spatial, and consequently temporal, signature. Because of the resonant behavior of nanorods, these evanescent modes are now able to be converted to propagating modes due to Purcell effect^41^. According to the finiteness of our structure in the $x-y$ plane, the supported eigenmodes are quantized and this limits the resolution of the proposed structure^30^.

In order to show the validity of the conversion of evanescent modes to propagating modes, we perform a three-dimensional simulation. A small Hertzian dipole is placed on top of one of the nanorods and the medium is excited with a broadband 10-fs pulse centered at 550 nm, whose bandwidth covers the smallest-scale modes, which present the highest wave numbers. We record the received signal in the far field of the medium until *t* = 140 fs. As depicted in Fig. 2b many resonant peaks are exhibited in comparison to the emission without the lens. If we use the Fourier transform to obtain the spectrum of this signal, it confirms the excitation of evanescent modes and their conversion to propagating ones. In other words, this structure shows a dispersive bahaviour due to the Fabry–Perot-like resonance, and a phase shift at reflection happens at the two ends of the structure along the *z*-direction^39^. This dispersion relation is well below the light line, so that the sub-wavelength modes are now allowed to be excited inside the lens and propagate through transverse directions. This capability enables us to perform the desired sub-wavelength lensing.

## Results

In the previous section we showed that due to the dispersion, the sub-wavelength information carried by decaying waves are stored in the temporal signature of the structure. Here, We will show how one can exploit this property to achieve super-resolution focusing and, reciprocally imaging, from the far field using time-reversal technique.

### Time-reversal sub-wavelength focusing

To demonstrate the super-resolution property of the structure in the focusing scheme, in the first step we must record the wavefield propagated from an infinitesimal source located at the top of the nanorods in the far field. In fact, we record the Green’s function of this propagation channel. To that aim, we use a set of four optical transducers in the far field of the structure, as depicted in Fig. 2a. Here, the time-reversal technique is used, in which, we flip the recorded Green’s functions in time, and re-emit them into the medium. In this method, each specific position on top of a nanoparticle has a unique temporal signature which is the coherent-summation of all supported modes of the proposed medium associated with that position.

**Figure 3:**
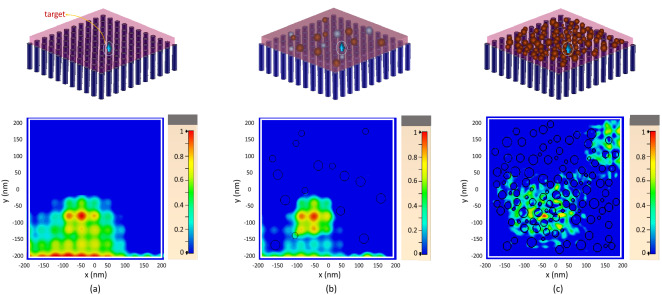
Super-resolution property of the structure in the focusing scheme inside tissue samples with different scatterer densities of (**a**) zero (homogeneous medium), (**b**) 2% and (**c**) 20%. Tissue sample on top of the nanorod lens (top) and normalized field distribution inside tissue (bottom).

To evaluate the capability of the proposed aluminum nanorod lens for focusing the light inside biological samples with a resolution beyond the diffraction limit, we performed full wave simulations (as described in Methods). In the first setup, a Formalin-Fixed Paraffin-Embedded (FFPE) sample of breast tissue with a thickness of 50 nm is located on top of the lens (Fig. 3a). The refractive index behaviour of this lossy sample in the visible region is obtained with Bolin and Preuss^42^. Assume we want to focus the light inside this sample on top of the nanorod positioned at $(x=$ − 40 nm , $y=$ − 80 nm). Once the Green’s functions between this position and four directions in far field (positions of transducers) are known, we play these signals backward in time from the transducers simultaneously. The focal plane of the structure is at *z*=25 nm, at the center of the sample. Recording the fields in this plane, Fig. 3a demonstrates the maximum in time of the squared magnitude of the received fields on each pixel. We can see that based on the Full-Width at Half-Maximum (FWHM) criterion, a $\lambda /7$ wide spot is obtained using the proposed structure which is beyond the $\lambda /2$ diffraction limit.

**Figure 4:**
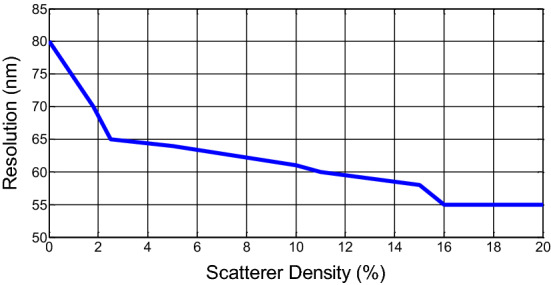
Focusing resolution versus scatterer density inside the tissue sample.

Now, the sub-wavelength focusing ability of this lens using time-reversal technique is demonstrated. Refractive-index inhomogeneities cause the light to be strongly scattered inside the biological medium. To model the light propagation inside a scattering medium, we study the focusing inside lossy samples with numerous random scatterers inside. Here, the host samples contain some bubble-like scatterers made out of different materials with different indexes and sizes located at random positions, so that, during the propagation, the light will be reflected, absorbed, diffracted and scattered and we believe that this can model an inhomogeneous and disordered multiple scattering medium. The rest of the simulation procedure is the same as we did in the previous part. Figure 3b and c show the two samples with low (2%) and high (20%) scatterer densities. The focused fields show that the resolution in these two cases are about $\lambda /8$ and $\lambda /10$ respectively. These results are better than the case without scatterers. Furthermore, we see that clearer field distributions with lower undesired hot spots are obtained. This demonstrates that scatterers can be beneficial for the focusing when the time-reversal technique is used. This observation is also consistent with researches in microwave range, showing that an increase in multiple scattering effects leads to a higher focusing resolution of the time-reversed signals^33,34^ so that, the time-reversal technique is a good choice for manipulating waves in complex and scattering media^36^. To show this effect, we perform more simulations with samples with different scatterer densities and the focusing resolutions for each density are summarized in Fig. 4. As expected from the time-reversal focusing property, the interaction between wave and scatterers increases the multipath propagation which leads to a larger numerical aperture. So that according to FWHM criterion, the focusing resolution was improved from 80 nm ($=\lambda /7$) to 55 nm ($=\lambda /10$). Also, it can be seen that after density of 16%, the resolution does not improve anymore. The reason for this lies in the fact that although the spatiotemporal degrees of freedom increases for higher densities, the proposed metalens cannot compensate for the relative losses experienced by these higher order modes. So, we estimate that our limiting resolution to be about 55 nm.

### Super-resolution imaging

**Figure 5:**
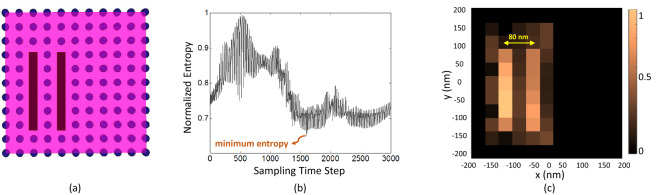
Imaging of two parallel bars inside tissue (**a**) input medium (**b**) minimum entropy concept to obtain the optimum time of imaging (**c**) reconstructed image.

We demonstrated that our lens can focus light on 55 nm wide spots. Keeping in mind that the property of far field sub-wavelength focusing is based on the conversion of evanescent modes to propagating modes, we leverage this capability for the imaging problem. To that aim, we place a tissue sample consisting of two parallel bars having a 40% higher refractive index than the tissue in one side of the sample (Fig. 5a). Our goal is to image these two objects and separate between them within the distance of 80 nm inside the tissue. The imaging operation is as follows: as stated in the focusing scheme, we assume that we know the 4$\times$121 transient Green’s functions $G_{en}(t)$ in which, *e* is the index of each of the four receiving transducers and *n* represents the nanorod index where the source is located. With this knowledge, which could be called a calibration step, we can obtain 121 distinct pixels in the reconstructed image. For each imaging step, we emit a pulse from one direction and record the scattered fields received at four optical transducers positions. We repeat this emission and recording for the other three directions, so that now we have a set of 4$\times$4 signals $O_{re}(t)$ (*e* corresponds to the emitting direction and *r* corresponds to recording direction). The image reconstruction is based on a cross-correlations procedure which is often done in ultrasonography. We numerically focus on emission and reception on the top of each rod, which is a pixel of the image. Using this method, the reconstruction operation for the *n*-th pixel becomes:

1 $$\begin{aligned} I_n(t)=\sum _{r=1}^{4}\sum _{e=1}^{4}\underbrace{(\overbrace{O_{re}(t) *_t G_{en}(-t)}^\text {emission step})*_t G_{rn}(-t)}_\text {reception step} \end{aligned}$$

where $*_t$ is the convolution in time operator.

In conventional interferometry measurement techniques, the value of *n*-th pixel in the image is the maximum in time of $I_n(t)$ signal. But this is valid when we have sufficient number of sources and detectors. For example, in^30^, Lemoult and Fink numerically used eight recorders and transmitters around a silver metalens and performed polychromatic interferometric far field super-resolution imaging. However, the structure is bulky and difficult to realize. Here, we used only four optical transducers to reduce the duration of the imaging procedure and the complexity of the structure. Therefor due to data loss, the maximum in time does not reconstruct the accurate image anymore. Instead, we propose using the local minimum entropy criterion as suggested by reference^43^ and we apply it to our imaging procedure. The formula to obtain the entropy is given in (2). The plot of the entropy over time is depicted in Fig. 5b.

2 $$\begin{aligned} R(I)=\frac{[\sum _{n=1}^{121}(I_n(t))^2]^2}{\sum _{n=1}^{121}(I_n(t))^4} \end{aligned}$$

A discussion on the use of entropy criterion to obtain the optimum time slice is as follows: this technique was originally used in electromagnetic compatibility applications to locate electromagnetic interference sources^44,45^. In our method, this criterion is applied to reconstruct images. According to the curve in Fig. 5b, the global minimum corresponds to the instant at which the back-propagated waves focus back to the nanorods and contains the most information for obtaining the image. Once this time slice has been found using (2), we reconstruct the image on top of each nanorod (each pixel in the image) according to the $I_n$ signal at this time (which is 1594-th time step in the imaging presented in Fig. 5). To the best of our knowledge, this is the first time that the entropy criterion is used for a time-reversal imaging system in reconstruction step.

Figure 5c clearly shows two distinct bars inside tissue so we can estimate the imaging resolution of our lens to be about 80 nm.

**Figure 6:**
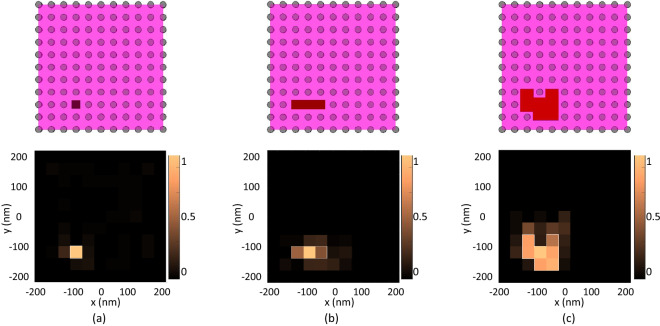
Input medium (top) and result of (bottom) imaging of diffusive material inside tissue at three steps with different shapes and material with a refractive index of (**a**) 1.5, (**b**) 1.4 and (**c**) 1.2 times the tissue.

We also consider another scenario for the far field imaging of an object spreading over time. Assume we have a diffusive material like nanomedicine or a cell and we want to image the spreading pattern of this material inside a tissue. We image this in three steps and in each step the refractive index of the object is 1.5, 1.4 and 1.2 times the refractive index of FFPE sample. Results are given in Figs. 6a–c. Again, it is shown that a resolution of 80 nm is obtained. Also, we see that the lens has the capability of imaging objects inside tissue with a 20% higher refractive index which is due to sub-wavelength information of the object translated in the spectrum of the field generated inside the structure.

## Discussion

It is known that properties of available materials play a main role in the study of structures in the optical range. To take advantage of recent advances in fabrication technology, we proposed here the use of nanorods made of aluminum. However, it is important to discuss about other possibilities. We changed the metal and repeated the numerical focusing procedure for gold, copper, silver and Perfect Electric Conductor (PEC). (Properties are obtained from Ref.^38^). As shown in Table 1, the resolutions are not changed significantly compared to aluminum. The reason lies in the behaviour of our lens. As explained before, each eigenmode of the structure has the capability of carrying an amount of energy. The higher the order of the mode, the weaker the coupling of the mode to the far field. On the other hand, this higher order mode has the chance of existing inside the structure for a longer time which is called *lifetime*. According to the Purcell effect^41^, this mode has a higher ability to carry energy and in this way, the weak coupling to the far field is compensated. Therefore, higher order modes which carry deeper sub-wavelength informations do not suffer much from losses and have almost the same chance of propagation as lower order modes. In conclusion, although the intrinsic loss of the material shortens the life of higher order modes and decreases the resolution, the latter is mainly determined by the number of supported modes in the lens.

**Table 1:** Resolution obtained by different materials.

Material	Resolution
Aluminum	0.13$\lambda$
Gold	0.13$\lambda$
Copper	0.12$\lambda$
Silver	0.12$\lambda$
PEC	0.11$\lambda$

Next we investigated the effect of increasing the number of rods. We changed *N* from 11 to 15 and 21 and got a resolution of 65 nm ($=0.12\lambda$) and 60 nm ($=0.11\lambda$) respectively, which, as expected, is enhanced due to the increase in the number of supported modes. Theoretically, it is shown that in an ideal infinite wire media, the resolution is only determined by the spacing between elements^28^. As we know, the dimensions of a real biological sample are in micrometer scale, so for a practical superlensing scenario we can easily expand the number of rods in the transverse plane in order to cover the samples and expect a higher resolution due to the increase in the number of supported modes. However, in this work we considered nanometer sizes because of the limited computational resources.

Another point that should be discussed is the effect of the spatiotemporal degrees of freedom. It has been proven that unlike other methods such as frequency hopping or coherent control of optical field that exploit only temporal or spatial degree of freedom, the time-reversal technique takes advantage of both simultaneously^46^. This is also the main reason why we used the polychromatic approach instead of the monochromatic one which helps us to reduce the number of transducers required for experimental setups. Hence, we believe that according to recent achievements in the spatiotemporal control of light, the realization of a practical setup is quite possible. For instance, one can use Optically Addressed Spatial Light Modulators (OASLMs)^47^ as transducers. This enables to control the light in millions of pixels. Also, the proposed lens could be integrated with existing optical microscopes to perform an unprecedented super-resolution imaging inside tissue.

Finally, we discuss the effect of the noise on the imaging resolution of our proposed plasmonic resonant metalens. In an experimental case, there are two main sources of noise during the imaging procedure. The measurement of the Green’s functions and the measurement of the far field scattered signals in the image reconstruction step. As explained, the imaging is based on the knowledge of a set of Green’s functions. Using many averaging procedures to obtain the Green’s functions precisely, and this needs to be done only once, we believe that the main source of error does not come from the knowledge of these Green’s functions. However, the other source will deteriorate the image. For a similar time-reversal imaging procedure in Ref.^30^, it is discussed that if we manage to record the transient fields with a noise level lower than 1% (which is a reasonable assumption), the imaging procedure gives a super-resolved image of the object. It is noteworthy that recently introduced active convolved illumination techniques^48^ based on optical amplification and using auxiliary sources can significantly improve the spectral signal-to-noise ratio and go beyond this noise threshold. Also, using filters in reconstruction algorithms, introduced in atmospheric imaging^49^ in the presence of noise and scattering, could increase the robustness of our algorithm to the noise and lead to some level of additional detail in the obtained image.

## Methods

Full wave simulations were performed using time-domain solver of CST Microwave Studio. To obtain the Green’s functions between nanorod and transducers, we put a z-directed discrete port on top of each nanorod and record the propagated fields in the far field using electric field probes. The excitation signal is a 10-fs Gaussian pulse centered at 550 nm and the duration of record is 140 fs. For the focusing scheme, we simply flip the recorded signal at each direction in Matlab and illuminate the lens with four plane wave sources simultaneously. We probe the near field on top of the nanorods and the maximum in time of the squared amplitude of the received fields on each position is presented in Fig. 3. For the imaging scheme, we preform a four-step procedure. At each step, a short pulse from one of far field directions illuminates the combination of lens and object, and the scattered field is recorded at four directions. Using Eqs. (1) and (2), this $O_{er}(t)$ signal in conjunction with the set of Green’s functions reconstructs the images depicted in Figs. 5 and 6. In practice, to obtain higher resolution, we replace the set of $O_{re}(t)$ by $O_{re}(t) - O^{0}_{re}(t)$, where $O^{0}_{re}(t)$ is the scattered fields in each direction without the object.

## Supplementary Information

Supplementary Information.
